# The Enhanced Catalytic Performance and Stability of Rh/γ-Al_2_O_3_ Catalyst Synthesized by Atomic Layer Deposition (ALD) for Methane Dry Reforming

**DOI:** 10.3390/ma11010172

**Published:** 2018-01-22

**Authors:** Yunlin Li, Jing Jiang, Chaosheng Zhu, Lili Li, Quanliang Li, Yongjie Ding, Weijie Yang

**Affiliations:** 1 School of Chemistry and Chemical Engineering, Zhoukou Normal University, Zhoukou 466001, China; yunlinli06@163.com (Y.L.); czhu2016@163.com (C.Z.); liquanliang04@hotmail.com (Q.L.); yding35@gmail.com (Y.D.); wyang291@gmail.com (W.Y.); 2 School of Computer Science and Technology, Zhoukou Normal University, Zhoukou 466001, China; jjiangedu@163.com; 3 School of Life Science and Agriculture, Zhoukou Normal University, Zhoukou 466001, China

**Keywords:** heterogeneous catalyst, atomic layer deposition, crystal growth, XPS, X-ray techniques, microstructure

## Abstract

Rh/γ-Al_2_O_3_ catalysts were synthesized by both incipient wetness impregnation (IWI) and atomic layer deposition (ALD). The TEM images of the two catalysts showed that the catalyst from ALD had smaller particle size, and narrower size distribution. The surface chemical states of both catalysts were investigated by both XPS and X-ray Absorption Near Edge Structure (XANES), and the catalyst from IWI had higher concentration of Rh^3+^ than that from ALD. The catalytic performance of both catalysts was tested in the dry reforming of methane reaction. The catalyst from ALD showed a higher conversion and selectivity than that from IWI. The stability testing results indicated that the catalyst from ALD showed similar stability to that from IWI at 500 °C, but higher stability at 800 °C.

## 1. Introduction

To deal with climate change, most countries are making efforts to reduce the emission of carbon dioxide (CO_2_), which is the major cause for greenhouse effects. Dry reforming of methane (DRM) (${\mathrm{CH}}_{4}+{\mathrm{CO}}_{2}\overset{\mathrm{catalyst}}{\to }2\mathrm{CO}+2H_{2}$) is an effective way to make use of CO_2_ to pure valuable chemical products and slow down the global warming [1,2,3,4,5,6]. The product of syngas, a mixed gas of H_2_ and CO, could be used for Fischer–Tropsch reaction in chemical industry, to synthesize various chemical compounds and liquid hydrocarbons [7,8,9,10,11,12]. The energy in the liquid phase can not only increase the safety for storage and transportation, but also increase the energy density. 

Catalysts play an important role in DRM reaction. The conventional preparation methods for these catalysts include incipient wetness impregnation (IWI), precipitation, and colloidal synthesis [13,14,15]. Recently, atomic layer deposition (ALD) has also been used to synthesize heterogeneous catalysts [16,17,18,19,20,21]. Even though ALD is mainly used for thin film deposition, depositing noble metals on oxide surface results in a nucleation delay because of slow nucleation kinetics. Moreover, together with the tendency of metal nanoparticles agglomeration, nanoparticles are formed on the oxide surface [19]. Compared with conventional catalyst preparation methods, ALD brings many benefits, such as monodispersed size, uniform purity, less contaminations, and the ability to penetrate highly porous supports [19]. ALD has been widely used to deposit noble metals—including Pt, Ru, and Pd—on various metal oxide supports by using organic based metal precursors [22,23]. When the ALD cycle is less than 10, it usually generates nanosized noble metal particles with an average diameter less than 2 nm [23]. These small sized nanoparticles could bring extra benefits to the catalytic reaction, such as less side reactions and higher catalyst stability [19].

ALD has been used to synthesize Ni/SiO_2_ and Ni/TiO_2_ catalysts for DRM reaction [24,25]. Compared with transition metals, noble metals could provide many advantages for catalytic reactions. Noble metals—such as Pt, Rh, Ir, and Ru—are highly active towards DRM reaction and are more resistant to carbon formation than transition metals [26,27,28,29]. Rh/γ-Al_2_O_3_ catalyst has been widely used for DRM reaction in previous reports [12]. However, its major synthesis method has been focused on IWI. To the best of our knowledge, Rh/γ-Al_2_O_3_ catalyst has never been synthesized by ALD in previous reports. In this research, Rh/γ-Al_2_O_3_ catalysts were synthesized by both IWI and ALD, and their catalytic performance was compared and tested in DRM reaction. 

## 2. Experimental

Catalyst preparation: The support of γ-Al_2_O_3_ (Sigma-Aldrich, acid type, ~0.25 mm, New Jersey, the USA) was annealed at 500 °C for 2 h before catalyst preparation. Rh(acac)_3_-O_3_-H_2_ were used as the precursors for Rh deposition. The deposition was carried out at 250 °C and 300 mTorr, respectively. O_3_ was produced with an ozone generator from O_2_ (99.999%). After each step of precursor exposure (0.5 s), N_2_ (99.999%) was injected into the chamber for purging (40 s). As a comparison, the Rh/γ-Al_2_O_3_ catalyst was also prepared by IWI. An appropriate amount of Rh(NO_3_)_3_∙6H_2_O (Sigma-Aldrich, 36% wt % Rh) was dissolved in DI H_2_O, and the solution volume equaled the total pore volume of the γ-Al_2_O_3_. Rh solution was then dropped into γ-Al_2_O_3_. After drying, the catalyst was calcined in air at 500 °C for 4 h, and finally reduced at 500 °C for 2 h in H_2_ environment. The mass percentage of Rh in both catalysts was 5%.

Catalyst characterization: The as-synthesized catalysts from both IWI and ALD are characterized with transmission electron microscopy (Hitachi, X250, Japan) for loaded Rh nanoparticle size, size distribution, and lattice fringe. The surface chemical states and valance composition of metal particles are characterized by X-ray photoelectron spectroscopy (XPS, Kratos, UK), and X-ray absorption near edge structure (XANES, Philips, the USA respectively. The crystal structure is characterized by X-ray diffractometer (XRD, Bruker D8, Bruker, Germany). The specific surface area was measured by Brunauer–Emmett–Teller (BET) method with a micromeritics instrument (Tokyo HT-190, Japan), and the relative pressure is controlled to be 0.01 to 1. 

**Figure 1:**
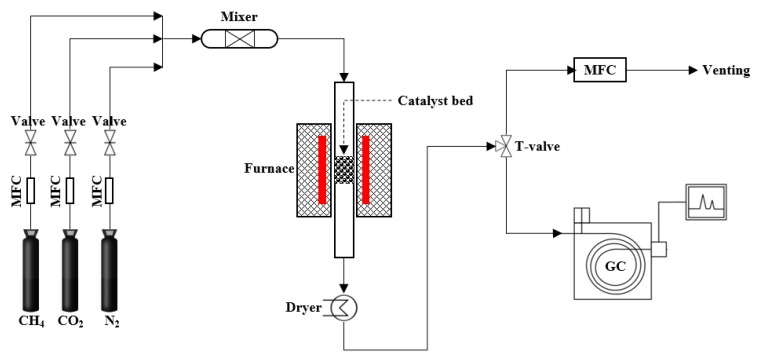
The methane dry reforming reaction and analysis system.

Catalyst testing: Both catalysts were tested in DRM reaction, which was carried out in a continuous-flow quartz fixed-bed reactor (internal diameter = 10 mm, length = 400 mm) under atmospheric pressure, and the reaction temperature was controlled between 100–1000 °C, and the catalyst mass was 0.1 g. The flow rates of feeding gases (CH_4_, CO_2_, and N_2_) were controlled to be 20, 20, and 5 mL/min, respectively. The furnace heating rate was 1 °C/min. The effluent gases were analyzed by a gas chromatograph (HP6890, Hewlett Packard, Japan) equipped with a fused silica capillary column and thermal conductivity detector. The reaction system was simplified in Figure 1. 

## 3. Results and Discussion

**Figure 2:**
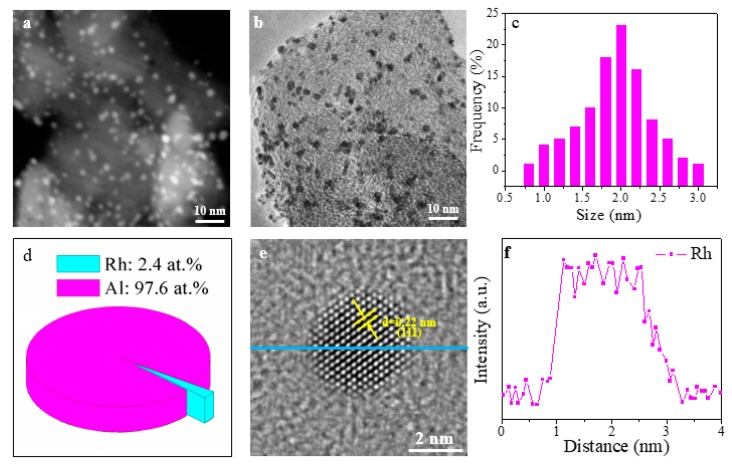
TEM characterization of Rh/γ-Al_2_O_3_ catalysts synthesized by ALD: (**a**) STEM image; (**b**) TEM image (**c**) Rh size distribution; (**d**) Rh/Al atomic ratio based on the EDX data; (**e**) HRTEM image; (**f**) EDX spectrum.

**Figure 3:**
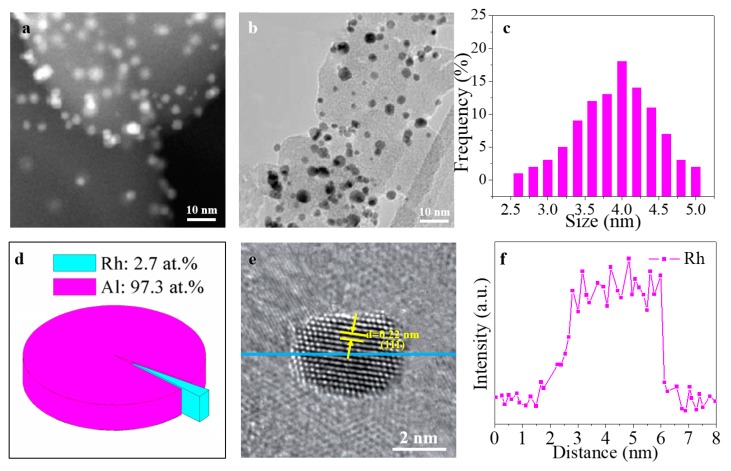
TEM characterization of Rh/γ-Al_2_O_3_ catalysts synthesized by IWI: (**a**) STEM image; (**b**) TEM image; (**c**) Rh size distribution; (**d**) Rh/Al atomic ratio based on the EDX data; (**e**) HRTEM image; (**f**) EDX spectrum.

The TEM characterization of Rh/ γ-Al_2_O_3_ catalysts from both ALD and IWI is shown in Figure 2 and Figure 3. Figure 2a and Figure 3a shows the scanning transmission electron microscopy (STEM) images, and the bright dots refer to the Rh nanoparticles. The bright field TEM images are presented in Figure 2b and Figure 3b. Both STEM and TEM images have confirmed that Rh nanoparticles are homogeneously distributed in γ-Al_2_O_3_ in of Rh/γ-Al_2_O_3_ catalysts from both ALD and IWI. The size distribution of Rh nanoparticle size is shown in Figure 2c (ALD), and Figure 3c (IWI), the corresponding the average sizes are 1.8 and 4.2 nm and the standard deviations are 0.43 and 0.5, respectively, for the catalysts from ALD and IWI. This indicates that the catalyst from ALD shows smaller particle size and narrower size distribution than that from IWI. It is known that, when the mass of Rh loaded on γ-Al_2_O_3_ is the same, the smaller particle size could provide a larger surface area. The molar ratio of Rh to Al in both as-synthesized catalysts based on the energy dispersive X-ray (EDX) data is shown in Figure 2d and Figure 3d, the Rh atomic percentage in the catalysts from ALD and IWI is 2.4% and 2.7%, respectively. Considering the variation, it is safe to say that the two catalysts have the same atomic percentage of Rh. The HRTEM images of the two catalysts are shown in Figure 2e and Figure 3e. The lattice distance of 0.22 nm corresponds to (111) plane in Ru crystal structure. Moreover, the clear fringe in the HRTEM images indicates that the Rh nanoparticles are well crystalized. The corresponding energy dispersive EDX spectrum is shown in Figure 2f and Figure 3f, which clearly demonstrates the existence and purity of Rh nanoparticles. The TEM characterizations in Figure 2 and Figure 3 have clearly showed that metal phased Rh nanoparticles have been formed in the catalysts synthesized from both methods. However, the ALD method is superior to IWI in narrowing the size and size distribution of Rh nanoparticles.

**Figure 4:**
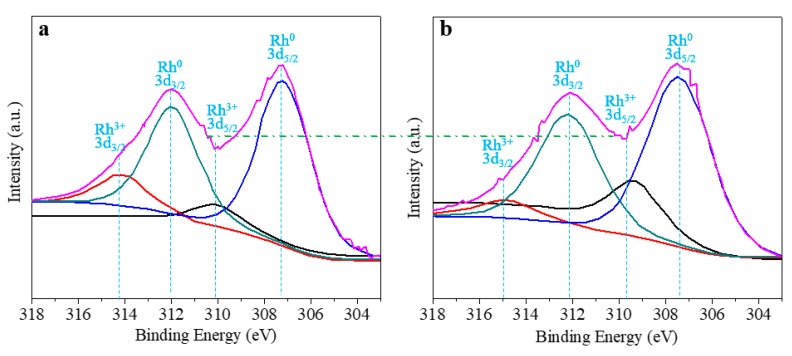
(**a**) XPS of Rh/γ-Al_2_O_3_ catalyst from ALD; (**b**) XPS of Rh/γ-Al_2_O_3_ catalyst from IWI.

The surface chemical states of Rh nanoparticles in the catalysts are investigated by XPS. Accordingly, the XPS spectra of Rh/γ-Al_2_O_3_ catalysts from ALD and IWI are shown in Figure 4a,b, respectively. It is noted that Rh 3d_3/2_ and Rh 3d_5/2_ at 312 and 307.4 eV are the characteristics of Rh^0^. Both spectra are dominated by these two peaks, indicating that Rh^0^, the active site for DRM, is presented in both catalysts. The Rh 3d_3/2_ and Rh 3d_5/2_ at ~314 and ~310 eV are the characteristics of Rh^3+^. Due to the peak overlap, these two peaks cannot be clearly presented. However, the valley between Rh^0^ 3d_3/2_ and 3d_5/2_ in Figure 4a is lower than that in Figure 4b, indicating that the catalyst from ALD has a lower Rh^3+^ concentration than that from IWI. The reason could be explained as follows. In ALD synthesis, Rh^0^ is directly synthesized from the reaction, and a post reduction is not needed. However, in IWI synthesis, Rh_2_O_3_ (Rh^3+^) was formed in a transient step, and followed by a post reduction to reduce Rh_2_O_3_ (Rh^3+^) in to Rh (Rh^0^). Due to the limitations in thermodynamic equilibrium, it cannot achieve a complete Rh_2_O_3_ reduction [30]. Therefore, the ALD synthesis is more beneficial to form metal phased Rh (Rh^0^) than the IWI synthesis. 

**Figure 5:**
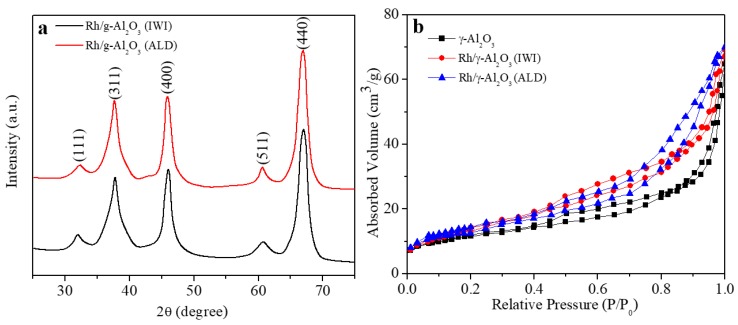
(**a**) XRD patterns of Rh/γ-Al_2_O_3_ catalysts from ALD and IWI; (**b**) N_2_ adsorption/desorption isotherm curves of γ-Al_2_O_3_ powders, and Rh/γ-Al_2_O_3_ catalysts from ALD and IWI.

The crystal structure of the catalysts could be determined by X-ray diffraction (XRD). The XRD patterns of both catalysts shown in Figure 5a are in a good agreement with the standard JPCDS card (NO. 10-0425) [31], which is attributed to the support of γ-Al_2_O_3_. The XRD patterns of Rh nanoparticles are not shown in the two catalysts. This could be because the loaded mass of Rh nanoparticles in both catalysts is lower than the detection limit of XRD, and similar results were reported in many previous reports [11,12,13]. Another possibility for the absence of Rh peaks in the XRD patterns is that, as shown in Figure 2a and Figure 3a, the size of Rh nanoparticles is very small (~2–4 nm). Therefore, their peak broadening is so pronounced that no sufficient diffraction can occur to result in resolved diffraction peaks. As discussed above, the existence of Rh^0^ has been confirmed by TEM and XPS. The specific surface area is one of the most important parameters for catalysts, as a larger specific surface area could potentially provide more active reaction sites for catalytic reactions. The specific surface area is widely characterized in the laboratory by Brunauer–Emmett–Teller (BET) method. The BET is conducted with the relative pressure range between 0.01 and 1. Before the BET measurement, the catalysts were degassed at 120 °C for 12 h. According to the N_2_ adsorption and desorption curves in Figure 5b, the specific surface area of γ-Al_2_O_3_, Rh/γ-Al_2_O_3_ (ALD), and Rh/γ-Al_2_O_3_ (IWI) is calculated as 128, 131 and 119 m^2^/g, respectively. Considering the sample variation, it could be generally concluded that the catalyst preparation process brings little impact on the specific surface area of the catalysts. The catalysts synthesized by ALD and IWI show similar specific surface area. Therefore, the BET surface area is not the key factor for their different catalytic performance.

**Figure 6:**
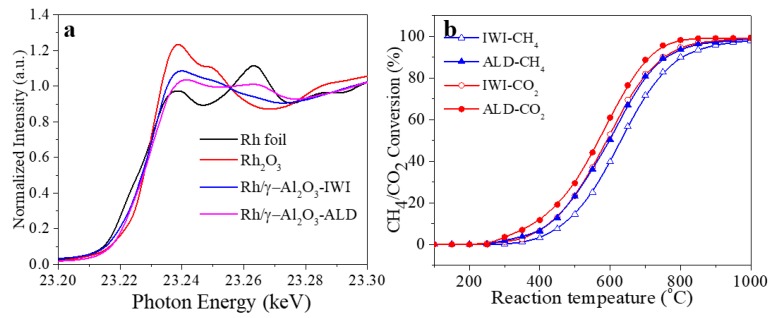
(**a**) Rh K-edge XANES spectra for Rh foil, Rh_2_O_3_, Rh/γ-Al_2_O_3_ catalysts; (**b**) CH_4_ and CO_2_ conversion of Rh/γ-Al_2_O_3_ catalysts from IWI and ALD between 100–1000 °C.

The normalized X-ray absorption near edge structure (XANES) is a useful technique to qualitatively determine the valance composition of metal particles. The XANES spectra of both Rh/γ-Al_2_O_3_ catalysts are shown in Figure 6a. The XANES intensity of Rh foil represents for characteristics of Rh^0^, while Rh_2_O_3_ represents the characteristics of Rh^3+^. By comparison, it is found that both catalysts contain Rh^3+^, and Rh/γ-Al_2_O_3_ (IWI) has a higher level of oxidation than that from ALD, which is consistent with the results shown in the XPS spectra, and the corresponding reason has been explained above. In the DRM reaction, the catalytically active sites are Rh^0^, instead of Rh^3+^ [32,33]. Therefore, a higher concentration of Rh^0^ means a higher catalytic performance. 

The catalytic performance of Rh/γ-Al_2_O_3_ catalysts from both IWI and ALD was investigated in DRM reaction, and testing conditions for both catalysts have been kept consistent. The conversion and selectivity of the reaction are defined as

(1) $$X_{{\mathrm{CH}}_{4}}=(F_{{\mathrm{CH}}_{4}\left(\mathrm{in}\right)}-F_{{\mathrm{CH}}_{4}\left(\mathrm{out}\right)})/F_{{\mathrm{CH}}_{4}\left(\mathrm{in}\right)}\times 100\%$$

(2) $$X_{{\mathrm{CO}}_{2}}=(F_{{\mathrm{CO}}_{2}\left(\mathrm{in}\right)}-F_{{\mathrm{CO}}_{2}\left(\mathrm{out}\right)})/F_{{\mathrm{CO}}_{2}\left(\mathrm{in}\right)}\times 100\%$$

(3) $$S_{H_{2}}=F_{H_{2}\left(\mathrm{out}\right)}/\left[2\times (F_{{\mathrm{CH}}_{4}\left(\mathrm{in}\right)}-F_{{\mathrm{CH}}_{4}\left(\mathrm{out}\right)}\right)]\times 100\%$$

(4) $$S_{\mathrm{CO}}=\frac{F_{\mathrm{CO}\left(\mathrm{out}\right)}}{\left(F_{{\mathrm{CH}}_{4}\left(\mathrm{in}\right)}-F_{{\mathrm{CH}}_{4}\left(\mathrm{out}\right)})+(F_{{\mathrm{CO}}_{2}\left(\mathrm{in}\right)}-F_{{\mathrm{CO}}_{2}\left(\mathrm{out}\right)}\right)}\times 100\%$$

X, S, and F stand for conversion, selectivity, and gas flow rate, respectively. The gas flow rates of CH_4_, CO_2_, H_2_, and CO in the outlet are calculated with their peak areas in the gas chromatography. Together with the inflow rates of CH_4_ and CO_2_, the gas conversion and selectivity are calculated. The conversion of CH_4_ and CO_2_ is shown in Figure 6b. It can be seen that, between 300–900 °C, the catalyst synthesized by ALD shows a higher conversion rate of both CH_4_ and CO_2_ by 10% than that by IWI. According to the methane dry reforming reaction (CH_4_ + CO_2_ → 2CO + 2H_2_), the conversion of CH_4_ and CO_2_ is supposed to be the same. However, due to the possible side reactions (such as, CO + 2H_2_ → CH_4_ + H_2_O, CH_4_ + H_2_O → CO + 3H_2_, etc.), the conversion of CH_4_ and CO_2_ is presented to be different in Figure 6b.

**Figure 7:**
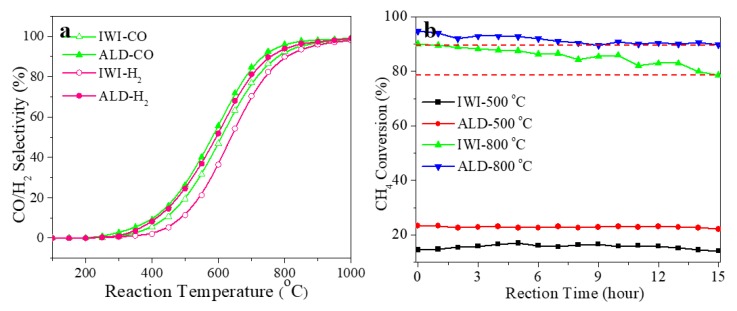
(**a**) CO and H_2_ selectivity of Rh/γ-Al_2_O_3_ catalysts from IWI and ALD between 100–1000 °C; (**b**) CH_4_ conversion of Rh/γ-Al_2_O_3_ catalysts from IWI and ALD over 15 h under 500 and 800 °C.

The selectivity of CO and H_2_ from both catalysts are shown in Figure 7a. The catalyst synthesized by ALD shows higher selectivity in both CO and H_2_. Moreover, both the conversion and selectivity data in Figure 6b and Figure 7a show that the reaction under two different catalysts have a similar ignition temperature of ~250 °C. However, after ignition, the catalyst synthesized by ALD generally shows a higher catalytic performance than that synthesized by IWI below 900 °C. This indicates that, to achieve the same conversion and selectivity, the catalyst synthesized by ALD requires a lower temperature that that by IWI, and achieves a higher energy efficiency. However, when the temperature exceeds 900 °C, the conversion and selectivity of the two catalysts become similar. This could be attributed to the DRM reaction reaching thermodynamic equilibrium and/or mass transfer being the rate determining factor, and the catalysts cannot change the reaction behaviors. Combined with the characterization provided above, the enhanced conversion (CH_4_ and CO_2_) and selectivity (CO and H_2_) in the temperature range of 300–900 °C could be attributed to the different size, size dispersion, and oxidation level of Rh nanoparticles in the Rh/γ-Al_2_O_3_ catalysts synthesized by ALD and IWI.

The stability of the two catalysts are investigated by running the DRM reaction for 15 h, and the conversion of CH_4_ is monitored. The reaction is run at 500 and 800 °C, respectively, and the corresponding results are shown in Figure 7b. It can be seen that the change of CH_4_ conversion of the two catalysts at 500 °C is negligible in 15 h, indicating that both of them have a relatively high stability at this temperature. However, when the temperature is further increased to 800 °C, the conversion of CH_4_ from ALD catalyst is reduced by ~5.0%, and while that from IWI is reduced by ~11.5%, in 15 h of reaction. Therefore, it is concluded that the catalyst synthesized by ALD shows a higher thermal stability at a relatively high temperature over that synthesized by IWI.

**Figure 8:**
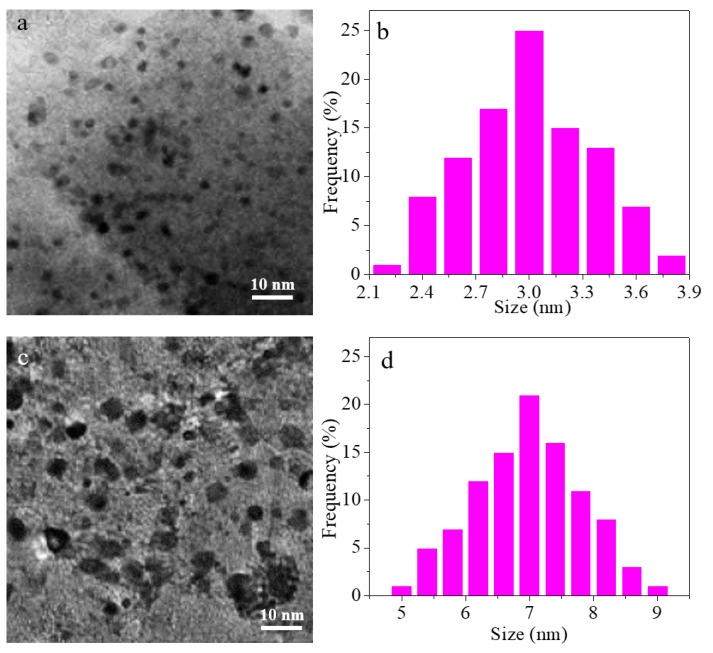
(**a**) TEM image of Rh/γ-Al_2_O_3_ catalysts (ALD) after running 15 h in DRM reaction; (**b**) Size distribution of Rh nanoparticles in Figure 8a; (**c**) TEM image of Rh/γ-Al_2_O_3_ catalysts (IWI) after running 15 h in DRM reaction; (**d**) Size distribution of Rh nanoparticles in Figure 8c.

As shown in Figure 7b, the CH_4_ conversion is decreased in both catalyst under 800 °C for 15 h. To investigate this change, the TEM images of both catalysts after running at 800 °C for 15 h are displayed in Figure 8a,c, respectively, and the corresponding size distribution is shown in Figure 8b,d. It can be seen that the average Rh particle size is increased from 1.8 to 2.8 nm in Rh/γ-Al_2_O_3_ catalysts (ALD), while the average size is increased from 4.2 to 7.3 nm in Rh/γ-Al_2_O_3_ catalysts (IWI). Therefore, sintering occurs in both catalysts. The EDX investigation on both catalysts did not detect obvious carbon residues in the catalysts, which was consistent with previous research results that indicated that noble metal catalysts are more resistant to carbon formation [26]. Therefore, the major reason for the decreased CH_4_ conversion in DRM reaction could be attributed to Rh nanoparticle sintering. 

## 4. Conclusions

Rh/γ-Al_2_O_3_ catalysts were synthesized by ALD and IWI. The catalyst from ALD had a smaller size and narrower size distribution of Rh nanoparticles. The XPS and XANES showed that Rh^0^ was presented in both catalysts, but the catalyst from IWI had a higher level of Rh^3+^. DRM reaction testing showed that the catalyst from ALD had higher conversion and selectivity in the range of 300–900 °C. Both catalysts had similar stability at 500 °C, the catalyst from ALD had a higher stability at 800 °C. The research in this paper shows that the catalyst synthesized by ALD shows higher catalytic performance and stability over the commonly used IWI method.
